# Laboratory Indicators of Aggrecan Turnover in Juvenile Idiopathic Arthritis

**DOI:** 10.1155/2016/7157169

**Published:** 2016-01-27

**Authors:** Katarzyna Winsz-Szczotka, Kornelia Kuźnik-Trocha, Katarzyna Komosińska-Vassev, Agnieszka Jura-Półtorak, Krystyna Olczyk

**Affiliations:** Department of Clinical Chemistry and Laboratory Diagnostics, Faculty of Pharmacy with the Division of Laboratory Medicine, Medical University of Silesia, Ulica Jedności 8, 41-200 Sosnowiec, Poland

## Abstract

*Objectives*. Evaluation of chondroitin sulfate (CS), as an early marker of aggrecan degradation, and chondroitin sulfate 846 epitope (CS846), as a biomarker of CS synthesis, is an attempt at answering the question whether the therapy used in juvenile idiopathic arthritis (JIA) patients contributes to the normalization of biochemical changes in aggrecan.* Methods and Results*. Serum levels of CS and CS846 as well as catalase (CT), superoxide dismutase (SOD), and glutathione peroxidase (GPx) activities in erythrocyte were assessed in patients before and after treatment. In the course of JIA, aggrecan metabolism is disturbed, which is reflected by a decrease (*p* < 0.001) in CS serum level and an increase (*p* < 0.05) in CS846 concentration. Furthermore, increased (*p* < 0.001) activities of CT, SOD, and GPx in untreated JIA patients were recorded. The anti-inflammatory treatment resulted in the normalization of CS846 level and SOD and GPx activities. In untreated patients, we have revealed a significant correlation between serum CS and CS846, CT, CRP, ESR, MMP-3, and ADAMTS-4, respectively, as well as between CS846 and CT, GPx, CRP, ESR, and TGF-*β*1, respectively.* Conclusion*. The observed changes of CS and CS846 in JIA patients indicate a further need of the therapy continuation aimed at protecting a patient from a possible disability.

## 1. Introduction

Articular cartilage is an avascular hypocellular hyaline connective tissue that has a limited capacity for self-repair. Once damaged by mechanical trauma or pathology, it is highly susceptible to structural degradation, making it particularly difficult to restore [1–4]. This tissue destruction often progresses spatiotemporally from the articular surface to the subchondral bone and leads to the loss of zonal tissue architecture [4, 5]. This loss is attributed primarily to the disorder of both the synthesis and the degradation of structural components within the extracellular matrix (ECM), including type-II-collagen fibrils and a proteoglycan, aggrecan. It is believed that aggrecan degradation is an early and reversible process, whereas the breakdown of the collagen network is irreversible, contributing to the loss of joint function [3]. Thus, an early therapy, which is preceded by appropriate diagnostics, can prevent irreversible changes in the osteoarticular system. The attempts to find early biomarkers to determine the severity and/or progression of joint damage in children with juvenile idiopathic arthritis (JIA) are especially important. Too late or wrong diagnosis of arthritis in children may result in a disability shortening life expectancy. Such biomarkers may be fragments of aggrecan that are released into biologic fluids during its turnover [6, 7].

Aggrecan is a multimodular molecule expressed by chondrocytes. Its core protein is composed of three globular domains and a large extended region, for glycosaminoglycan (GAG) chains attachment, that is, chondroitin sulfates (CS, up to 90%) and keratan sulfates [5, 6, 8]. Due to negative charge of GAG's chains, in particular CS, aggrecan is able to hydrate the collagen network and provides cartilage with its properties of compressibility and elasticity [1, 5]. Maintenance of the extent of CS side chain substitution in aggrecan is therefore critical for the function of the articular cartilage [6, 8]. Moreover, these GAGs modulate the cellular behaviours including proliferation, differentiation, and migration during development and diseases [4, 5, 7]. Any increase or decrease in a degradation rate and/or biosynthesis of CS may have pathological consequences and lead to the development of JIA. While the excessive activities of proteolytic enzymes and glycosidases seem to play the biggest role in aggrecan degradation, reactive oxygen species (ROS) attack is also an important degradative agent [3, 6, 7]. The thesis of the intensified enzymatic degradation of PGs/GAGs is confirmed by significantly higher concentrations of MMP-3 (metalloproteinase-3) or ADAMTS-4 (a disintegrin and metalloproteinase with thrombospondin motifs 4), found in blood of JIA patients, which was reported in our earlier research [7, 9]. The prooxidative-antioxidative imbalances constitute yet another pathogenic factor, which leads to aggrecan metabolism disturbances in JIA children [8, 10]. Both core proteins of PGs and GAGs chains are susceptible to oxidative damage stimulated by the excess of ROS [8, 11]. On the other hand, antioxidant properties of CS have been observed. This antioxidant activity is due to the chelation of transition metal ions, which simultaneously limits endogenic biosynthesis of ROS [12, 13]. Due to the above-mentioned processes, aggrecan degradation products occur in the patient's blood, and they are free CS chains or the chains of GAGs bound to core protein fragments.

Catabolic processes of ECM components, which take place in the cartilage, are probably not balanced by the biosynthesis of these compounds. This assumption seems to be confirmed by elevated concentrations of factors which stimulate ECM biosynthesis, that is, a transforming growth factor-beta (TGF-*β*1) or platelet-derived growth factor-BB (PDGF-BB), which were assayed in the blood of JIA patients [9].

Taking into consideration the above-mentioned relations, the present quantitative evaluation of both CS, as an early marker of aggrecan degradation, and chondroitin sulfate 846 epitope (CS846), as a biomarker of aggrecan synthesis, is an attempt at answering the question whether the therapy used in JIA patients, whose purpose is to reduce the inflammatory and immunological parameters of the disease activity, simultaneously contributes to the normalization of biochemical changes in aggrecan and thus inhibits the dysfunction of articular cartilage. Moreover, considering antioxidative CS effects and the presence of oxidative stress in JIA patients, the aim of the study was to determine the relation of CS and CS-846 with levels of the most important enzymes for detoxification of peroxides in living cells, that is, catalase, superoxide dismutase (SOD), and glutathione peroxidase (GPx) determined in the blood of patients with newly diagnosed JIA and in the same patients after therapy modifying inflammation.

## 2. Materials and Methods

### 2.1. Subject

The clinical study was carried out on the blood obtained from 30 children (19 girls, 11 boys), aged from 2 to 15 years (mean 7.27  ±  4.49), with newly diagnosed JIA, and 30 individuals with the same age. The patients were diagnosed and classified according to the International League of Associations for Rheumatology (ILAR) criteria as oligoarthritis or polyarthritis. Moderate disease activity was assessed in all patients, using Juvenile Arthritis Disease Activity Score. The exclusion criteria constituted other forms of JIA according to the ILAR criteria, as well as any other chronic and autoimmune diseases. Moreover, the accuracy of diagnosis was confirmed by laboratory tests, namely, by determining indicators of inflammatory responses, that is, C-reactive protein (CRP, immunonephelometric assay) and erythrocyte sedimentation rate (ESR, Westergren technique), measuring rheumatoid factor (RF, latex enhanced immunoturbidimetric test), and determining the titre of antinuclear antibodies (ANA, indirect immunofluorescence assay) (Table 1).

Presented in Table 1, results of MMP-3, ADAMTS-4, TGF-*β*1, and PDGF-BB were obtained in our earlier investigations [7, 9] using immunoenzymatic methods.

The tests were repeated on the same patients after therapy, when the clinical outcome improved (11.60 ± 0.21 months after the beginning of the therapy). The clinical improvement was determined by 30 ACR Pediatric criteria. The applied treatment was based on stable doses of nonsteroidal anti-inflammatory drugs, oral glucocorticoids (at maximum dose of 1 mg/kg/day of prednisone, with gradual dose reduction), and methotrexate (≤15 mg per square meter of body-surface-area once a week).

Informed consent was obtained from teenage participants under the age of 16 according to the ethical guidelines of the Helsinki Declaration. For all underage patients, parents or legal guardians signed the informed consent. The Local Ethics Committee of the Medical University of Silesia approved the research protocol used in this study. No conflicts of interest have occurred during implementation and completion of the study.

### 2.2. Biochemical Analysis

Venous blood samples were collected after overnight fasting and placed into tubes without anticoagulant and heparin-treated tubes. In the first case, serum was obtained after centrifugation and used for assessment of the CS and CS846 levels whereas, after centrifugation, plasma was removed from samples collected in heparin-treated tubes but remaining erythrocytes were washed three times with 0.9% NaCl and lysed in 1 : 1 (v/v) of double-deionised water. Erythrocyte lysates were used for determination of catalase, superoxide dismutase, and glutathione peroxidase activity as well as hemoglobin concentrations. Serum samples and erythrocyte lysates were stored at −80°C until needed for biochemical analyses.

The determination of one parameter was completed within one day; consequently the interassay variation was insignificant.

#### 2.2.1. Isolation and Purification of Serum CS

Blood glycosaminoglycans are comprised of GAGs chains of native proteoglycans and GAGs derived from the degradation of both blood and tissue PGs. CS represents the major GAGs in circulation, and their isolation from serum was conducted with the use of methods developed by Volpi et al. [14] and Olczyk et al. [15]. GAGs were released from serum PG core proteins by alkaline treatment after extensive papain digestion. Papain was added to the serum and samples were allowed to remain for 24 h at 60°C in a stirrer. After boiling, the mixture was brought to pH 9.0 by adding NaOH. Following the storage for 24 h at 40°C, the mixture was neutralised with trichloroacetic acid (TCA). Mixture was centrifuged and the pellet was washed with TCA. The precipitate was discarded. To the pooled supernatants, 3 volumes of ethanol was added, and GAGs were allowed to precipitate for 12 h at 4°C. After centrifugation, aqueous CH_3_COOK was added to the precipitate. The obtained GAG solution was treated with 3 volumes of ethanol. GAGs were allowed to precipitate for 12 h at 4°C. Following centrifugation, precipitate was dissolved in H_2_O and GAGs were isolated by precipitation after the addition of cetylpyridinium chloride (CPC). After incubation and centrifugation, GAGs precipitated by CPC were finally washed with C_2_H_5_OH containing NaCl and centrifuged once more. The supernatants were discarded and a final precipitate (isolated and cleared serum GAGs) was stored at −80°C until the use for a biochemical analysis. The total amount of GAGs, including CS, was determined by the hexuronic acid assay according to the Blumenkrantz and Asboe-Hansen [16] method, with analytical sensitivity of 0.5 mg/L and calibration range from 0.5 to 50 mg/L. The coefficient of intra-assay variation was less than 6%.

Hexuronic acids concentration, as a measure of CS concentration, was determined both prior to and after the use of agents specifically degrading glycans other than CS. CS concentration was determined as the difference of obtained values.

Thus, dermatan sulfate was removed from isolated GAGs samples using depolymerisation with chondroitinase B (from* Flavobacterium heparinum*); then, the mixture of heparinase I and heparinase III (both from* Flavobacterium heparinum*) was added to a reaction medium in order to remove heparin sulfate/heparin. Digestion was carried out according to the manufacturer's instructions. All enzymes were obtained from Sigma Aldrich (USA).

#### 2.2.2. The Assay of the Concentration of CS846 in Serum Samples

CS846 level was measured using blindly tested coded serum samples, in duplicate. The tested compound was quantitatively measured using an* Aggrecan Chondroitin Sulfate 846 Epitope Elisa* test kit provided by IBEX Pharmaceuticals Inc. (Montreal, Canada), according to the manufacturer's instructions. The minimal detectable concentration of this compound was 20.00 ng/mL. The intra-assay variation of the CS846 levels was less than 6%.

#### 2.2.3. The Assay of the Concentration of Antioxidant Defense System Activity

To assess antioxidant defense system activity, the samples were assayed for catalase, superoxide dismutase, and glutathione peroxidase activities.

Catalase activity was determined by measuring the decomposition of hydrogen peroxide at 240 nm, according to the method of Aebi [17], and expressed in Bergmeyer units/g of hemoglobin. The coefficient of intra-assay variation was less than 7.5%.

Superoxide dismutase activity was measured using the kit of Randox Superoxide Dismutase (Ransod), supplied by Randox Laboratories (United Kingdom), according to the manufacturer's protocol. The appropriate Test Ransod Control was used as a quality control test. The coefficient of intra-assay variation was less than 6.2%.

Glutathione peroxidase activity was measured using the kit of Randox Glutathione Peroxidase (Ransel), supplied by Randox Laboratories (United Kingdom), according to the manufacturer's protocol. For the control of precision, Test Ransel Control was used. The coefficient of intra-assay variation was less than 4.2%.

Hemoglobin was measured using a commercially available kit.

### 2.3. Statistical Analysis

A statistical analysis was carried out using Statistica 10.0 package (StatSoft, Cracow, Poland). The normality of distribution was verified with the Shapiro-Wilk test. The data obtained were expressed as mean values and standard deviation. Since the variables were normally distributed, the parametric Student's* t*-test was used to evaluate the differences between untied variables. To compare the same parameters in each patient, before the treatment and after the restoration of clinical improvement, the paired Student's* t*-test was used. Pearson's correlation coefficient was employed for the statistical analysis of correlations between two variables. *p* values of less than 0.05 were considered significant.

## 3. Results

The results are presented in Table 2. Based on the obtained results, in patients with untreated JIA, we found a significant reduction of serum concentrations of CS, quantified by the hexuronic acid assay. As shown in Table 2, the untreated patients had a 51% lower (*p* < 0.001) level of these GAGs, compared to the controls. It was observed that the therapy modifying the course of inflammation, which was employed in JIA patients, resulted in a significant increase (*p* < 0.001) in serum levels of CS in these patients. However, treated JIA patients still had a markedly lower (*p* = 0.04) serum level of the CS than the control subjects. When compared to the control values, the mean decrease in CS level was by 15%.

Low concentration of CS, recorded in serum of JIA patients with untreated arthroplasty, was negatively statistically significantly correlated with the concentrations of laboratory inflammatory markers, that is, CRP and ESR. The obtained values were as follows: CS and CRP (*r* = −0.59, *p* = 0.014) and CS and ESR (*r* = −0.36, *p* = 0.028), respectively. We recorded insignificant relationships between CS and CRP (*r* = −0.11, *p* = 0.097) as well as CS and ESR (*r* = −0.05, *p* = 0.226) in patients with JIA whose clinical condition had stabilized.

Since the metabolism of cartilage CS is associated with the activity of proteolytic enzymes, we decided to assess the relationship between serum CS level and MMP-3 and ADAMTS-4. A correlation analysis revealed that in the untreated JIA patients there was a significant negative correlation between CS level and MMP-3 (*r* = −0.63, *p* = 0.019) and ADAMTS-4 (*r* = −0.40, *p* = 0.029) levels, respectively. Corresponding correlations were not found in treated JIA patients (CS and MMP-3 (*r* = −0.16, *p* = 0.118); CS and ADAMTS-4 (*r* = 0.30, *p* = 0.087)).

A quantitative assessment of CS846 revealed significantly higher (*p* = 0.013) levels of this parameter in the serum of untreated JIA patients than in the control group. Compared to the control values, the mean increase in serum CS846 was by 52%. Anti-inflammatory treatment led to a significant decrease (*p* = 0.006) in this marker concentration versus pretreatment situation. The blood concentration of CS846 in treated children with JIA corresponded to its concentration in the control group.

Correspondingly to the above-described relations between CS and inflammatory markers, there was a negative correlation between CS846 and these markers only in the group of children with untreated JIA. In untreated patients, the correlations were as follows, CS846 and CRP (*r* = −0.51, *p* = 0.008) and ESR (*r* = −0.44, *p* = 0.031), respectively, whereas in treated patients the following correlations were recorded: CS846 and CRP (*r* = 0.23, *p* = 0.305) as well as CS846 and ESR (*r* = −0.17, *p* = 0.056), respectively.

Due to the fact that CS846 blood concentration reflects the size of aggrecan synthesis, we decided to evaluate the relation between CS846 and TGF-*β*1, and PDGF-BB too. We recorded significant relationships between CS846 and TGF-*β*1 (*r* = 0.49, *p* = 0.011), but insignificant relationship between CS846 and PDGF-BB (*r* = 0.15, *p* = 0.261) in untreated patients with JIA. Furthermore, no correlation was recorded between CS846 and TGF-*β*1 (*r* = −0.09, *p* = 0.311) as well as PDGF-BB (*r* = 0.34, *p* = 0.461), respectively, in treated JIA patients.

To assess antioxidant defense system activity, the samples (red blood cells) obtained from the control and JIA patients were assayed for catalase (CT), superoxide dismutase (SOD), and glutathione peroxidase (GPx) activities. The analysis of the enzyme activity showed that in the course of untreated JIA, significantly increased catalase, superoxide dismutase, and glutathione peroxidase activities were observed. The mean increase in CT, SOD, and GPx activities was by 62% (*p* < 0.001), 38% (*p* < 0.001), and 41% (*p* < 0.001), respectively, versus the control values.

It was also found that the employed therapy, which resulted in suppressing inflammation, simultaneously contributed to a significant decrease (*p* < 0.001) in all antioxidant enzyme activities in red blood cells versus pretreatment situation. While the activity of SOD and GPx was the same as the enzyme activity in the controls, the activity of CT was still significantly higher (*p* = 0.006) than in healthy children, contributing 121% of this value.

A correlation analysis, whose results are presented in Table 3, revealed that in the untreated JIA patients there was a significant positive correlation between serum CS level and CS846 level (*r* = 0.67, *p* < 0.001) as well as significant positive correlation between serum CS level and CT activity (*r* = 0.57, *p* = 0.001). No correlation was recorded between this GAG's serum level and SOD (*r* = −0.08, *p* = 0.664) and GPx (*r* = −0.25, *p* = 0.072) activities, respectively. Corresponding correlations were found in the same patients after symptoms improvement due to an anti-inflammatory therapy. Thus, in treated JIA patients serum CS level correlated significantly with CS846 (*r* = −0.61, *p* < 0.001), CT (*r* = 0.60, *p* < 0.001), and GPx (*r* = 0.40, *p* = 0.034), but not with SOD (*r* = 0.23, *p* = 0.226), respectively. As shown in Table 3, in untreated JIA patients, CS846 correlated with CT (*r* = 0.43, *p* = 0.011) as well as GPx (*r* = 0.18, *p* < 0.001) but not with SOD (*r* = −0.39, *p* = 0.278), respectively. Corresponding correlations were not found in treated JIA patients or in controls (Table 3).

## 4. Discussion

At present, no single biomarker has been validated as an indicator of joint damage in the course of JIA. Despite the fact that chondroitin sulfates, as the main components of aggrecan, could function as markers of cartilage damage in patients with JIA, it cannot be considered as a specific indicator of cartilage turnover due to its presence in other tissues. Our studies showed that CS serum levels in children with newly diagnosed arthropathy were significantly lower than the concentration of these GAGs both in the same children when clinical improvement was observed and in healthy controls. Although early steps of cartilage ECM degradation process should be reflected by the increase in blood GAG levels, it seems that degradation taking place over a prolonged time may result in gradual decrease in serum GAGs pool. We have concluded that the concentration of CS in blood of children with the arthropathy is directly related to the activity of both inflammatory processes and proteolytic degradation of ECM components. Recorded low concentrations of CS in untreated patients with JIA were significantly negatively correlated with inflammatory markers and with high plasma levels of MMP-3 and ADMTS-4 in these patients. It should be also mentioned that catabolism of ECM components is related to oxidation caused by ROS [11, 18]. In our study, complex evaluation of the antioxidant state in untreated JIA patients has shown a significantly increased activity of intracellular oxygen radical scavenging enzymes, that is, CT, SOD, and GPx, compared to the children from the remaining two groups. We suggest that the mentioned alterations are the sign of functional changes induced by radical overproduction. Of all evaluated free radical scavenging enzymes, catalase seems to have a special relation with CS alterations. Demonstrated significant correlation between this enzyme activity and CS blood level in untreated JIA patients appears to confirm the participation of these compounds in the formation of the body response to developing oxidative stress, which promotes JIA manifestation. The changes of hydrogen peroxide seem to be the common denominator of the mentioned activity. On one hand, it is known that catalase prevents the accumulation of hydrogen peroxide. It is important due to the fact that hydrogen peroxide is a substrate of the Fenton reaction, which leads to generating the most reactive form of oxygen, namely, hydroxyl radical [18]. On the other hand, it is known that CS is able to chelate iron ions, which are catalysts of the above-mentioned reaction [12, 13]. In the effect of the chelation of iron ions, oxidative stress is reduced by eliminating the products of this reaction, and it is reasonable to hypothesize that blocking this reaction is more efficient than scavenging the reactive activity of the products formed. The next mechanism, common for the discussed compounds, which impedes the development of JIA, is related to the fact that several antioxidant compounds, including catalase or CS, have been found to be able to reduce NF-*κ*B activation with a consequent reduction in cartilage damage [19, 20]. The majority of cellular events involved in the inflammation process require a NF-*κ*B, a transcription factor that plays a central role in the transformation of cytokines, chemokines, or ROS, involved in the immune response [19–21].

When trying to explain aggrecan changes in children with JIA, we also demonstrated a significant increase in the serum level of CS846 [22–24]. The observed high concentration of the discussed biomarker, which is strongly related to CS concentration, seems to indicate a significant increase in anabolic processes of aggrecan, but not big enough to balance the degree of the proteoglycan degradation. Likewise, high concentration values of CS846 were recorded in the course of rheumatoid arthritis [23, 24]. It is also difficult to compare our results with those obtained in adults. Despite the clinical similarities between both diseases, the young age of patients with JIA could be the main factor responsible for the observed changes of different ECM components in blood. Our previous studies demonstrated that the plasma concentration of various GAG types, which reflects tissue alterations of ECM, is dependent on the age of the subject [25].

The factors which participate in modeling of ECM component synthesis are, among others, TGF-*β*1 and PDGF-BB [8, 9]. The concentration of both mentioned factors is significantly higher in the blood of patients with newly diagnosed JIA, which would confirm our earlier results [9]. However, only the concentration of TGF-*β*1 is statistically correlated with the CS846 level. This compound confirms the dependence between signaling paths related to TGF-*β* and cartilage elasticity, which is conditioned by aggrecan structure [26]. It is believed that mechanical stress activated latent TGF-*β* produced by cultured myofibroblasts. Moreover, the rigidity of the ECM also alters the response of cells to TGF-*β* stimulation via the phosphatidylinositol 3-phosphate/Akt signaling pathway [26, 27].

The evaluation of the relations between CS846 and antioxidative enzymes, which was carried out in this study, allowed us to find the relationship of the evaluated biomarker with the CT activity and its weaker relationship with GPx. Thus, we can conclude that an increase in the synthesis of CS is bigger in persons with higher antioxidative protection. This reconstruction is also greater in young patients with less intense inflammation, which is confirmed by the observed negative correlations between CS-846 and CRP and ESR. To answer the question of the influence of inflammation modifying treatment on cartilaginous aggrecan alterations were the aim of the study; however it is difficult to offer an unequivocal opinion. The employed therapy, which contributed to clinical improvement in patients, simultaneously did not result in complete normalization of changes in articular cartilage. This is our conclusion since we observed a lack of normalization of CS serum level in treated patients. A probable mechanism of such a state is related to autoimmune background of JIA. It is suggested that the core protein of aggrecan may be the first original target of autoreactivity [28]. Moreover, in the systemic circulation and in the synovial fluid of rheumatoid arthritis patients, the presence of GAG-specific antibodies was revealed [29].

## 5. Conclusion

In the course of JIA, the structure of cartilaginous aggrecan is disrupted, which is reflected by increase in CS846 and indirectly by a decrease in CS blood serum levels in patients. The anti-inflammatory therapy relieves the symptoms of JIA by decreasing both the pain and the intensification of inflammatory processes, although it does not lead to total regeneration of ECM elements, which are damaged by proteolytic-oxidative factors. The observed quantitative changes of CS in JIA patients indicate a further need of the therapy continuation aimed at protecting a patient from a possible disability.

## Figures and Tables

**Table 1:** Clinical characteristics of control subjects and JIA disease patients.

Parameter	Control subjects (*n* = 30)	Untreated JIA (*n* = 30)	JIA disease after attainment of clinical improvement (*n* = 30)
Age (years)	8.67 ± 4.12	7.27 ± 4.49	8.21 ± 4.01
Sex, female/male	17/13	19/11	19/11
JADAS-27	—	18 ± 8.56	4 ± 2.52
WBC (10^3^/*μ*L)	7.32 ± 2.19	10.04 ± 4.03^a^	7.14 ± 2.31^b^
RBC (10^6^/*μ*L)	4.95 ± 0.35	4.48 ± 0.41^a^	4.62 ± 0.36
Hb (g/dL)	13.85 ± 0.94	11.58 ± 1.38^a^	12.97 ± 1.16^a,b^
Ht (%)	40.96 ± 3.18	35.35 ± 3.61^a^	37.17 ± 7.39^a,b^
PLT (10^3^/*μ*L)	283.47 ± 73.83	405.47 ± 129.10^a^	351.20 ± 93.78^b^
Total cholesterol (mM)	4.12 ± 0.87	4.61 ± 1.42^a^	4.25 ± 1.65^b^
Glucose (mM)	4.56 ± 0.38	4.11 ± 1.16	4.40 ± 0.98^b^
Creatinine (*μ*M)	80.01 ± 12.66	52.97 ± 10.23^a^	64.54 ± 15.47^a,b^
CRP (mg/L)	1.24 ± 1.59	20.25 ± 24.00^a^	2.76 ± 0.57^b^
ESR (mm/h)	10.50 ± 7.03	42.00 ± 27.02^a^	13.11 ± 7.21^b^
ANA	—	56% (positive)	56% (positive)
RF	—	100% (negative)	100% (negative)
^*∗*^MMP-3 (ng/mL)	0.26 ± 0.13	2.25 ± 1.36^a^	0.55 ± 0.25^a,b^
^#^ADAMTS-4 (ng/mL)	15.55 ± 6.70	26.71 ± 15.17^a^	13.26 ± 8.10^b^
^#^TGF-*β* (ng/mL)	5.70 ± 1.93	8.13 ± 3.24^a^	3.88 ± 1.82^a,b^
^#^PDGF-BB (ng/mL)	1.19 ± 0.54	1.64 ± 0.75^a^	0.85 ± 0.49^a,b^

Results are expressed as mean ± SD; ^a^ *p* < 0.05 compared to control group; ^b^ *p* < 0.05 compared to untreated JIA patients; WBC, white blood cell; RBC, red blood cell; Hb, hemoglobin; Ht, hematocrit; PLT, platelet; CRP, C-reactive protein; ESR, erythrocyte sedimentation rate; ANA, antinuclear antibodies; RF, rheumatoid factor; MMP, matrix metalloproteinase; ADAMTS, a disintegrin and metalloprotease with thrombospondin motifs; TGF-*β*, transforming growth factor-beta; PDGF-BB, platelet derived growth factor-BB; ^*∗*^results reported in [7]; ^#^results reported in [9].

**Table 2:** The distribution pattern of aggrecan turnover markers and antioxidant defense system in control subjects and juvenile idiopathic arthritis patients.

Parameter	Control group (*n* = 30)	Nontreated JIA patients (*n* = 30)	JIA patients after treatment and attainment of clinical improvement (*n* = 30)
CS (hexuronic acids, mg/L)	87.00 ± 21.94	42.45 ± 13.35^b^	73.70 ± 15.53^a,d^
CS846 (ng/mL)	152.52 ± 79.72	231.96 ± 50.84^a^	165.88 ± 76.80^c^
CT (U Bergm/g Hb)	58.84 ± 4.51	95.05 ± 16.43^b^	70.98 ± 21.12^b,d^
SOD (U/g Hb)	759.36 ± 176.43	1049.08 ± 162.45^b^	720.65 ± 141.09^d^
GPx (U/g Hb)	33.95 ± 8.51	47.85 ± 6.51^b^	36.95 ± 6.62^d^

Results are expressed as mean ± SD; ^a^ *p* < 0.05 and ^b^ *p* < 0.001 compared to control group; ^c^ *p* < 0.05 and ^d^ *p* < 0.001 compared to untreated JIA patients.

JIA, juvenile idiopathic arthritis; CS, chondroitin sulfate; CS846, chondroitin sulfate 846 epitope; CT, catalase; SOD, superoxide dismutase; GPx, glutathione peroxidase.

**Table 3:** Correlation analysis between CS, CS846 and catalase, superoxide dismutase, and glutathione peroxidase (Pearson's correlation coefficients).

Parameter	CS	CS846
	*r* (*p*)	*r* (*p*)
Control group (*n* = 30)		
CS	—	0.29 (NS)
CT	0.26 (NS)	0.21 (NS)
SOD	0.11 (NS)	0.19 (NS)
GPx	0.15 (NS)	0.30 (NS)
Untreated JIA patients (*n* = 30)		
CS	—	0.67 (0.000)
CT	0.57 (0.001)	0.43 (0.011)
SOD	−0.08 (NS)	−0.39 (NS)
GPx	−0.25 (NS)	0.18 (0.000)
JIA patients after treatment (*n* = 30)		
CS	—	−0.61 (0.000)
CT	0.60 (0.000)	0.40 (NS)
SOD	0.23 (NS)	0.06 (NS)
GPx	0.40 (0.034)	−0.25 (NS)

JIA, juvenile idiopathic arthritis; CS, chondroitin sulfate; CS846, chondroitin sulfate 846 epitope; CT, catalase; SOD, superoxide dismutase; GPx, glutathione peroxidase; NS, not statistically significant.

## References

[1] Fox A. J. S., Bedi A., Rodeo S. A. (2012). The basic science of human knee menisci: structure, composition, and function. *Sports Health*.

[2] Wilusz R. E., Sanchez-Adams J., Guilak F. (2014). The structure and function of the pericellular matrix of articular cartilage. *Matrix Biology*.

[3] Mort J. S., Billington C. J. (2001). Articular cartilage and changes in arthritis: matrix degradation. *Arthritis Research*.

[4] Hayes A. J., Tudor D., Nowell M. A., Caterson B., Hughes C. E. (2008). Chondroitin sulfate sulfation motifs as putative biomarkers for isolation of articular cartilage progenitor cells. *Journal of Histochemistry and Cytochemistry*.

[5] Martel-Pelletier J., Kwan Tat S., Pelletier J.-P. (2010). Effects of chondroitin sulfate in the pathophysiology of the osteoarthritic joint: a narrative review. *Osteoarthritis and Cartilage*.

[6] Kiani C., Chen L., Wu Y. J., Yee A. J., Yang B. B. (2002). Structure and function of aggrecan. *Cell Research*.

[7] Winsz-Szczotka K., Komosińska-Vassev K., Kuźnik-Trocha K., Gruenpeter A., Lachór-Motyka I., Olczyk K. (2014). Influence of proteolytic-antiproteolytic enzymes and prooxidative-antioxidative factors on proteoglycan alterations in children with juvenile idiopathic arthritis. *Clinical Biochemistry*.

[8] Martel-Pelletier J., Boileau C., Pelletier J.-P., Roughley P. J. (2008). Cartilage in normal and osteoarthritis conditions. *Best Practice and Research: Clinical Rheumatology*.

[9] Winsz-Szczotka K., Komosińska-Vassev K., Kuźnik-Trocha K., Siwiec A., Zegleń B., Olczyk K. (2015). Circulating keratan sulfate as a marker of metabolic changes of cartilage proteoglycan in juvenile idiopathic arthritis; influence of growth factors as well as proteolytic and prooxidative agents on aggrecan alterations. *Clinical Chemistry and Laboratory Medicine*.

[10] Lipińska J., Lipińska S., Stańczyk J. (2014). Reactive oxygen species and serum antioxidant defense in juvenile idiopathic arthritis. *Clinical Rheumatology*.

[11] Parsons B. J. (2015). Oxidation of glycosaminoglycans by free radicals and reactive oxidative species: a review of investigative methods. *Free Radical Research*.

[12] Campo G. M., Avenoso A., Campo S. (2006). Purified human chondroitin-4-sulfate reduced MMP/TIMP imbalance induced by iron plus ascorbate in human fibroblast cultures. *Cell Biology International*.

[13] Egea J., García A. G., Verges J., Montell E., López M. G. (2010). Antioxidant, antiinflammatory and neuroprotective actions of chondroitin sulfate and proteoglycans. *Osteoarthritis and Cartilage*.

[14] Volpi N., Cusmano M., Venturelli T. (1995). Qualitative and quantitative studies of heparin and chondroitin sulfates in normal human plasma. *Biochimica et Biophysica Acta (BBA)—General Subjects*.

[15] Olczyk K., Głowacki A., Koźma E. M. (1997). Non-insulin-dependent diabetes mellitus-associated changes in serum glycosaminoglycans. *Pathophysiology*.

[16] Blumenkrantz N., Asboe-Hansen G. (1973). New method for quantitative determination of uronic acids. *Analytical Biochemistry*.

[17] Aebi H., Bergmayer W. U. (1974). Catalase. *Methods of Enzymatic Analysis*.

[18] Valko M., Leibfritz D., Moncol J., Cronin M. T. D., Mazur M., Telser J. (2007). Free radicals and antioxidants in normal physiological functions and human disease. *International Journal of Biochemistry and Cell Biology*.

[19] Campo G. M., Avenoso A., Campo S. (2008). Purified human plasma glycosaminoglycans reduced NF-kappaB activation, pro-inflammatory cytokine production and apoptosis in LPS-treated chondrocytes. *Innate Immunity*.

[20] Kobayashi Y., Nishikawa M., Hyoudou K., Yamashita F., Hashida M. (2008). Hydrogen peroxide-mediated nuclear factor *κ*B activation in both liver and tumor cells during initial stages of hepatic metastasis. *Cancer Science*.

[21] Simmonds R. E., Foxwell B. M. (2008). Signalling, inflammation and arthritis: NF-*κ*B and its relevance to arthritis and inflammation. *Rheumatology*.

[22] Jansen N. W. D., Roosendaal G., Lundin B. (2009). The combination of the biomarkers urinary C-terminal telopeptide of type II collagen, serum cartilage oligomeric matrix protein, and serum chondroitin sulfate 846 reflects cartilage damage in hemophilic arthropathy. *Arthritis and Rheumatism*.

[23] Rizkalla G., Reiner A., Bogoch E., Poole A. R. (1992). Studies of the articular cartilage proteoglycan aggrecan in health and osteoarthritis: evidence for molecular heterogeneity and extensive molecular changes in disease. *Journal of Clinical Investigation*.

[24] Conrozier T., Balblanc J.-C., Richette P. (2012). Early effect of hyaluronic acid intra-articular injections on serum and urine biomarkers in patients with knee osteoarthritis: an open-label observational prospective study. *Journal of Orthopaedic Research*.

[25] Komosinska-Vassev K., Olczyk P., Winsz-Szczotka K., Klimek K., Olczyk K. (2012). Plasma biomarkers of oxidative and AGE-mediated damage of proteins and glycosaminoglycans during healthy ageing: a possible association with ECM metabolism. *Mechanisms of Ageing and Development*.

[26] Wipff P.-J., Rifkin D. B., Meister J.-J., Hinz B. (2007). Myofibroblast contraction activates latent TGF-*β*1 from the extracellular matrix. *Journal of Cell Biology*.

[27] Horiguchi M., Ota M., Rifkin D. B. (2012). Matrix control of transforming growth factor-*β* function. *The Journal of Biochemistry*.

[28] Kamphuis S., Hrafnkelsdóttir K., Klein M. R. (2006). Novel self-epitopes derived from aggrecan, fibrillin, and matrix metalloproteinase-3 drive distinct autoreactive T-cell responses in juvenile idiopathic arthritis and in health. *Arthritis Research and Therapy*.

[29] György B., Tóthfalusi L., Nagy G. (2008). Natural autoantibodies reactive with glycosaminoglycans in rheumatoid arthritis. *Arthritis Research and Therapy*.

